# Are higher antidepressant plasma concentrations associated with fall risk in older antidepressant users?

**DOI:** 10.1007/s41999-022-00742-1

**Published:** 2023-01-19

**Authors:** A. C. Pronk, E. P. van Poelgeest, L. J. Seppala, K. J. Ploegmakers, B. H. Stricker, K. M. A. Swart, S. C. van Dijk, S. Oliai Araghi, L. C. P. G. M. de Groot, N. M. van Schoor, R. A. A. Mathôt, N. van der Velde

**Affiliations:** 1 Internal Medicine, Section of Geriatric Medicine, Amsterdam UMC location University of Amsterdam, Meibergdreef 9, Amsterdam, The Netherlands; 2 Aging and Later Life, Amsterdam Public Health, Amsterdam, The Netherlands; 3 Department of Epidemiology, Erasmus University Medical Center, Rotterdam, The Netherlands; 4 General Practice, Amsterdam UMC location Vrije Universiteit Amsterdam, Boelelaan 1117, Amsterdam, The Netherlands; 5 Department of Geriatrics, Franciscus Gasthuis & Vlietland, Rotterdam, The Netherlands; 6 Department of Internal Medicine, Erasmus University Medical Center, Rotterdam, The Netherlands; 7 Division of Human Nutrition and Health, Wageningen University, Wageningen, The Netherlands; 8 Epidemiology and Data Science, Amsterdam UMC location Vrije Universiteit Amsterdam, De Boelelaan 1117, Amsterdam, Netherlands; 9 Hospital Pharmacy–Clinical Pharmacology, Amsterdam UMC location University of Amsterdam, Meibergdreef 9, Amsterdam, The Netherlands

**Keywords:** Accidental falls, Antidepressants, Older adults, Pharmacokinetics, Adverse drug events, Plasma concentration

## Abstract

**Purpose:**

Antidepressants are well-established fall-risk increasing drugs (FRIDs) and therefore falls should be considered an important adverse drug event (ADE) of antidepressants. However, not all antidepressant users experience fall incidents and factors associated with increased fall risk among antidepressant users are incompletely understood. Our objective was to explore whether antidepressant plasma concentrations are associated with falls in older antidepressant users.

**Methods:**

For this study, we included antidepressant users of the multicenter B-PROOF study. Fall incidents were recorded prospectively using fall calendars. Antidepressant plasma concentrations were analyzed by Liquid chromatography-mass spectrometry (LC–MS) at baseline and at 2 years follow-up. The associations between the observed antidepressant concentration and fall risk were assessed using Cox proportional hazard and logistic regression models and adjusted for potential confounders.

**Results:**

In total 93 selective serotonin reuptake inhibitor (SSRI) and 41 antidepressant (TCA) users were identified. There was a significant association between baseline TCA plasma concentration and fall risk within users (HR 2.50, 95% CI 1.07–5.87, crude model). In the adjusted model, there were no significant associations between concentrations of SSRIs and fall risk.

**Conclusion:**

There might be an association between plasma concentrations of TCAs and the risk of falling in older users. However, these results needs to be interpreted with caution considering the small sample size and accompanying limitation of confinement to crude analyses. Therefore, these novel findings need to replicated in a larger cohort, preferably including adjustment for potential confounders and more frequent measures of plasma concentrations is needed.

**Supplementary Information:**

The online version contains supplementary material available at 10.1007/s41999-022-00742-1.

## Key summary points

**Aim:**

To explore the association between antidepressant plasma concentrations and fall risk in older users.

**Findings:**

An association was found between baseline TCA plasma concentration and fall risk within users. No association was found between the SSRIs plasma concentrations and fall risk.

**Message:**

The association between TCA plasma concentration and fall risk needs to be replicated before its relevance to clinical practice can be established.

**Supplementary Information:**

The online version contains supplementary material available at 10.1007/s41999-022-00742-1.

## Introduction

Falls in older adults are a major public health problem and associated with substantial health care costs and decreased quality of life [1]. Annually, one-third of individuals over the age of 65 falls at least once and 20% of these falls lead to severe injuries [2]. Well established fall risk factors are fall-risk-increasing drugs (FRIDs) [3] and antidepressants use has consistently been associated with increased fall risk [4]. Therefore, falls should be considered a common adverse drug event (ADE) of antidepressants [5]. However, not all antidepressant users fall and un(der)treated depression also increases fall risk [6, 7]. It is therefore important to identify for whom the risk–benefit ratio of antidepressant use is detrimental. Which individual factors in antidepressant users contribute to fall risk, however, is not completely understood. A dose–response relationship between antidepressants and the incidence of falls has been demonstrated in older adults [8, 9]. However, in older adults, there is substantial inter-individual variation in drug pharmacokinetics and dynamics [10, 11]. Also, often polypharmacy and multimorbidity are present, predisposing to high prevalence of drug–drug and drug–disease interactions [12]. This makes predicting (adverse) drug effects based on given dosage difficult. Therefore, the availability of antidepressant plasma concentrations could be of value in predicting fall risk in antidepressant users.

Measuring drug concentrations to guide dosing of medicines, optimize treatment response and minimizing ADE, is called therapeutic drug monitoring (TDM). Especially within psychiatry this is a widely used approach [13]. Whether antidepressant plasma concentrations is associated with fall risk, however, has not been studied before. Potentially antidepressant plasma concentration measurements could guide clinical decision making in falls prevention. For instance, with regard to decisions on deprescribing or exchanging the antidepressant to a safer alternative. Therefore, our objective was to explore whether fall risk in older adults is related to the antidepressant concentration in plasma, and whether plasma antidepressant concentrations could serve as a biomarker in clinical practice in falls prevention. We hypothesized that antidepressant users with higher exposure in plasma would have a higher risk of falling compared to those with lower concentrations.

## Methods

### Trial design and participants

For this explorative study, antidepressant users from the multicenter B-PROOF (B-Vitamins for the Prevention of Osteoporotic Fractures; ClinicalTrials.gov NCT00696514) study were included. A detailed description of the trial was published previously [14]. In short, B-PROOF was a large multicenter, double-blind, placebo-controlled trial for which persons 65 years and older with mildly elevated homocysteine levels were recruited to participate between 2008 and 2011, for a follow-up period of 2–3 years. A total of 2,919 participants were included. The primary aim was to assess whether the addition of vitamin B12 and folic acid to vitamin D therapy prevented osteoporotic fractures. Because the intervention did not affect fall-related outcomes, data could be used for the current observational study [15]. The Medical Ethics Committee of Wageningen University approved the study protocol of the B-PROOF study [14]. Before entering the study, all participants gave their written informed consent. All experiments were conducted in accordance with the principles of the Declaration of Helsinki.

### Antidepressant usage

Participants were considered antidepressant user based on pharmacy prescription and self-reported usage data. Based on the number of users, participants using TCAs: amitriptyline (N06AA09), nortriptyline (N06AA10), and the SSRIs: citalopram (N06AB04), escitalopram (N06AB10), fluoxetine (N06AB03), fluvoxamine (N06AB08), paroxetine (N06AB05), sertraline (N06AB06) and the serotonin-norepinephrine reuptake inhibitor (SNRI) venlafaxine (N06AX16) [16] were selected.

Usage was defined as having a prescription [based on pharmacy dispensing record data, obtained from the Dutch Foundation for Pharmaceutical Statistics (SFK)]. Participants having prescriptions up to 30 days prior blood withdrawal at baseline and/or follow-up visit were selected. To capture more potential users, also participants with a prescription of up to 30 days after the withdrawal date were selected as some participants might have not had a refill in the 30 days before. For both SSRIs and TCAs, this concerned two participants. In case of missing pharmacy data, usage was defined in case of self-reported usage based on questionnaires at baseline and follow-up.

To determine used dosage, defined daily dose (DDD) was used. DDD is a statistical measure of drug consumption and is the assumed average maintenance dose per day for a drug used for its main indication in adults [16]. The average prescribed daily dose was based on the prescription (or self-reported data) in closest proximity to the date of baseline blood sampling.

### Exposure: assessment of antidepressant concentration in plasma

Blood samples were obtained from participants in the morning. Participants were in a fasted state, or had had a light breakfast. Venous blood was drawn in an EDTA tube at both baseline and follow-up study visits, and stored at − 80 °C until analysis.

The plasma concentrations of the following antidepressants and their corresponding (active) metabolites were analyzed using LC–MS with electrospray ionization (Thermo Finnigan TSQ Access, Waltham, MA, USA): amitriptyline (and its metabolite nortriptyline), nortriptyline, citalopram (and its metabolite des(methyl)-citalopram), fluoxetine (and its metabolite norfluoxetine), fluvoxamine, paroxetine, venlafaxine (and its metabolite desmethylvenlafaxine) and sertraline (and its metabolite norsertraline). A detailed description of the analysis method can be found in the supporting information (Supplementary information Appendix S1). With regard to the antidepressants with (active) metabolites, for the analyses, in line with clinical practice, the sum was taken of amitriptyline, fluoxetine, venlafaxine and their respective active metabolites. Some amitriptyline and nortriptyline users had plasma concentration below the lower limit of quantification (LLQ) of the LC–MS method. In these samples, it was possible to determine semi-quantitative concentration values (Appendix S1).

### Outcome

The primary outcome was time to first fall during follow-up. Falls were defined as “an unintentional change in position resulting in coming to rest at a lower level or on the ground” as recommended by the Prevention of Falls Network Europe [17]. Falls were reported prospectively using fall calendars, which the participants filled in weekly and returned to the research team every three months. Participants were contacted via telephone in case of missing or unclear data. Participants were followed until their first fall incident until their drop-out date or the date of their last calendar, date of death or end of the study, which ever came first [14]. We used the data from fall calendars as a binary variable (yes/no) for the analyses of plasma concentrations at follow-up.

### Covariates

Participant data were collected at baseline and follow-up using a questionnaire that included data on age, gender, smoking, alcohol consumption, comorbidities and history of falls. During study visits, data were collected on (self-reported) medication use, body mass index (BMI), blood pressure, serum creatinine, physical performance, handgrip strength, cognitive status, depressive symptoms and presence of pain. For screening of depressive symptoms, the 15-item version of the Geriatric Depression Scale (GDS-15) was used. Cognitive performance was assessed by the mini-mental state examination (MMSE). For pain, the EURoQoL-item 4 was used (pain or other complaints). Baseline use of concomitant medication was grouped based on the ATC coding system, and polypharmacy was defined as usage of five or more medications [18]. Serum creatinine levels were used to calculate kidney function according to the Cockcroft and Gault formula.

### Statistical analyses

If antidepressant use was reported, and plasma concentration was undetectable, the respective concentration was set at half of the LLQ. For amitriptyline and nortriptyline, where also semi-quantitative concentrations below LLQ were available, half of the lowest detectable concentration was taken. The values of LLQ were fluoxetine: 20 µg/L, sertraline: 5 µg/L, paroxetine: 5 µg/L, (es)citalopram: 5 µg/L, amitriptyline: 22 µg/L and nortriptyline: 22 µg/L. An overview of the numbers with undetectable plasma concentrations can be found in Supplementary information Table S1. Because the number of participants in the various individual antidepressant groups were too low for the analyses, the individual antidepressants subclasses needed to be pooled into two groups: SSRIs and TCAs. Although venlafaxine is a selective serotonin-norepinephrine reuptake inhibitor (SNRI), we analyzed venlafaxine combined with the SSRIs. Since the dose of venlafaxine in our cohort did not exceed 150 mg (mg), SNRIs mainly inhibit reuptake of serotonin and thus closely resemble SSRIs [19].

Relationship between drug exposure and fall risk was analyzed with plasma concentrations expressed on both a continuous and categorical scale. If a participant used two or more antidepressants concomitantly, only the antidepressant with the highest concentration was included in the analyses. This concerned in total 4 participants at baseline and 1 at follow-up. First, the continuous plasma concentration levels of the individual antidepressants were analyzed. After that, we standardized the concentrations of the different antidepressants by creating *Z*-scores to be able to combine the continuous concentrations. Second, for each antidepressant, the median of the plasma concentration was calculated and the concentrations were reclassified into a binary category (below (which was set as reference) or above the median). Third, to explore the influence of the highest plasma concentrations, the antidepressant plasma concentrations were divided into four categories. The reference category contained the concentrations below the LLQ. After that, the concentrations above LLQ were equally divided into tertiles. For the TCAs, the remaining number of participants per category was too low. Thus the reference category contained the lowest tertile and the concentrations below LLQ and the middle and highest tertile were combined. The same was applied for the SSRI follow-up concentrations.

Baseline characteristics were compared between fallers and non-fallers in antidepressant users. Differences between groups were tested using Chi-square test, *t* test or a Mann–Whitney *U* test (categorical and continuous non-normally and normally distributed data, respectively). To study the association between the antidepressant plasma concentration at baseline and falls during follow-up, Cox proportional hazard models were used to calculate hazard ratios (HRs). First, we built a model adjusted for age and gender (model 1). Second, the following covariates were selected as potential confounders based on a Directed Acyclic Graph (DAG) and included in the models if they changed the HR of the association by ≥ 10% (model 2): region, BMI, smoking, alcohol, pain, depressive symptoms, MMSE, number of medication and estimated glomerular filtration rate (eGFR). If covariates could not be added, due to the lack of 10 fall events per covariate [20], only the unadjusted model is presented. For follow-up visit, logistic regression models were used to calculate odds ratios (ORs) for the association between concentration and fall risk prior to follow-up visit. The same models as described for the Cox proportional hazard models were applied.

To assess whether antidepressant plasma concentrations were correlated with dosage, we calculated Pearson correlation coefficients. Since there were no signs of multicollinearity (correlation coefficient < 0.5) between plasma concentration and dosage, as a possible marker for example for disease severity, was added to model 2 to test whether dosage could be a confounder.

*P* values < 0.05 were considered statistically significant. Statistical analyses were performed in SPSS (version 28.0, IBM, Armonk, NY, USA).

## Results

### Study population

A total of 132 antidepressant users were identified from the B-PROOF cohort. Of those, 59.8% reported a fall during follow-up. In Table 1, the baseline characteristics of fallers and non-fallers in antidepressant users are shown. Fallers were more often female, experienced more falls in the 12 months prior to study enrollment and more frequently used benzodiazepines concomitantly. The antidepressants most often used were paroxetine (34.6%) and amitriptyline (23.5%). The range of plasma concentrations were fluoxetine: < 20–366 µg/L (fluoxetine + norfluoxetine), fluvoxamine: 126–330 µg/L, sertraline: < 5–91 µg/L, paroxetine: < 5–370 µg/L, citalopram: < 5–174 µg/L, escitalopram: 24.4–28.6 µg/L, amitriptyline: < 22–77.7 µg/L (amitriptyline + nortriptyline), nortriptyline: < 22–329 µg/L. Median concentrations of the individual antidepressants did not differ significantly between fallers and non-fallers, both at baseline and follow-up (Supplementary information Table S2). In supplementary information Table S3 present an overview of the reference values for therapeutic and (potential) toxic concentrations. Paroxetine, (es)citalopram plasma concentrations were in therapeutic range, but relatively low. Sertraline and amitriptyline plasma concentration were below therapeutic range. Nortriptyline plasma concentrations were higher in fallers compared to non-fallers (non-significant) and at follow-up above therapeutic range.

**Table 1:** Baseline characteristics of antidepressant users

	Non-fallers	Fallers	*P* value*
	*N* = 53	*N* = 79	
Age (years)^a^	73.4 (5.7)	74.4 (5.9)	0.33
Gender^b^			0.02*
Male	22 (41.5)	18 (22.8)	
Female	31 (58.5)	61 (77.2)	
Body mass index (kg/m^2^)^a^	27.3 (4.0)	27.0 (3.6)	0.69
Alcohol use (yes)^b^	42 (79.2)	55 (69.6)	0.22
Smoking (current)^b^	12 (22.6)	13 (16.5)	0.67
Depressive symptoms (GDS)^c^	2 (1–3)	2 (1–5)	0.60
MMSE score^c^	28 (27–29)	28 (26–29.5)	0.71
Pain (yes)^b^			0.77
No pain	20 (37.7)	25 (31.6)	
Some pain	29 (54.7)	47 (59.5)	
Severe pain	4 (7.5)	7 (8.9)	
Physical performance^c#^	8 (4–10)	7 (4–9)	0.27
Handgrip strength maximum (kg)^a^	29.6 (9.7)	26.6 (9.1)	0.08
Walking aid use (yes)^b^	15 (28.3)	22 (27.8)	0.96
Number of falls in 12 months prior to study enrolment^b^			0.002*
No falls	31 (75.6)	26 (41.9)	
One fall	7 (17.1)	19 (30.6)	
Two or more falls	3 (7.3)	17 (27.4)	
eGFR (ml min^−1^ 1.73m^2^)^c^	70.0 (60.1–80.7)	65.3 (54.7–76.8)	0.19
Vitamin D (25OH) (nmol/l)^a^	53.6 (24.7)	53.2 (25.7)	0.92
Number of medications^a^	4.1 (2.4)	4.7 (2.3)	0.12
Polypharmacy (yes)^b^	12 (30)	28 (70)	0.12
Benzodiazepine use (yes)^b^	4 (7.5)	20 (25.3)	0.01*
SSRI use	37 (69.8)	56 (70.9)	0.89
TCA use	17 (32.1)	24 (30.4)	0.84
Opioid use (yes)^b^	2 (3.8)	10 (12.7)	0.08
Antipsychotic use (yes)^b^	4 (7.5)	5 (6.3)	0.79

Polypharmacy: use of five or more medications

*GDS* geriatric depression scale, *MMSE* mini-mental state examination, *eGFR* estimated glomerular filtration rate according to Cockcroft and Gault formula

*Statistically significant at *P* value < 0.05

^#^Physical performance: combined score of the walking test, chair stand test and tandem stand test (maximum = 12)

^a^Mean (SD)

^b^Presented as *n* (%)

^c^Median (IQR; 25–75%)

### SSRI concentration at baseline & time to first fall

The results of the association between plasma concentrations of SSRIs at baseline and time to first fall are presented in Table 2. There were no significant associations between baseline plasma concentration and time to first fall in SSRI users.

**Table 2:** Association between baseline SSRI plasma concentration and time to first fall

Category	*N*	Model 1^a^	*P* value*	Model 2^b^	*P* value*
Concentration divided on median	93	0.91 (0.54–1.55)	0.74	1.10 (0.63–1.94)	0.73
Concentrations below LLQ	10	Ref.	0.61		
Lowest tertile	26	1.59 (0.62–4.07)	0.33	–	
Middle tertile	29	1.16 (0.44–3.01)	0.77	–	
Highest tertile	28	1.05 (0.41–2.69)	0.92	–	
*Z*-score	93	0.96 (0.72–1.27)	0.76	1.09 (0.79–1.52)	0.59

Data are presented in Hazard ratio with 95% confidence interval

Number of events: 57

*N* number of plasma concentration

^a^Model 1: adjusted for age and gender

^b^Model 2 of median concentration and *Z*-score adjusted for DDD, other covariates did not influence the model. Model 2 of category percentiles was not applied considering the limited amount of events

*Statistically significant at *P* < 0.05

### SSRI concentration at follow-up visit and fall risk

The results of the association between plasma concentrations of SSRIs at follow-up and fall risk prior study visit are presented in Supplementary information Table S4. An increased fall risk was found for users with concentrations in the middle tertile compared to users with the lowest concentrations (OR 4.67, 95% CI 1.09–19.9). However, after adjusting for confounders this association disappeared.

### TCA concentration at baseline and time to first fall

The results of the association between plasma concentrations of TCAs and time to first fall are presented in Table 3. A significantly increased fall risk was seen for higher TCA plasma concentrations compared to the lowest plasma concentrations (HR 2.50 (1.07–5.87).

**Table 3:** Association between TCA baseline plasma concentration and time to first fall

	*N*	Crude model^a^	*P* value*
Concentration divided on median	41	1.50 (0.67–3.35)	0.32
Concentrations below LLQ and lowest tertile concentrations	31	Ref.	
Middle and highest tertile concentrations	10	2.50 (1.07–5.87)	0.04
*Z*-score	41	1.25 (0.86–1.81)	0.25

Data are presented in Hazard ratio with 95% confidence interval

Number of events: 24

*N* number of plasma concentration samples

*Statistically significant at *P* < 0.05

^a^Model 1 was not applied due to the limited amount of events

### TCA concentration at follow-up visit and fall risk

There were no significant associations between plasma concentrations at follow-up visit and fall risk prior to study visit (Supplementary information Table S5).

### Role of dosage

No dose–response association was found for the different antidepressant groups regarding fall risk in antidepressant users (Supplementary information Table S6).

## Discussion

Our results showed that the risk of falling in older users of TCAs is possibly associated with drug exposure, whereas no association was found in users of SSRIs. These results need to be interpreted with caution considering the small sample size and subsequently confinement to unadjusted analysis for the TCA users.

To our knowledge, this is the first study to assess whether antidepressant blood concentrations are associated with fall risk within antidepressant users in a relatively large cohort of older adults. Falls have been established to be an important ADE in community-dwelling older persons. Previous studies have consistently established the association between antidepressant use and falls [4]. However, literature linking antidepressant plasma concentrations and falls is scarce. A first indication that measuring antidepressant plasma concentrations might be of value in falls prevention was demonstrated in a case series of patients with drug-induced falls. This study suggested that TDM may help identify patients with drug-induced falls and confirm clinical suspicion of an ADE [21]. The observed possible association between TCA concentrations and fall risk is in line with earlier studies that addressed the role of antidepressant plasma concentrations in fall-related outcomes, e.g., orthostatic hypotension (OH) [22]. Significant correlations between serum concentrations of amitriptyline and fluvoxamine and orthostatic blood pressure drop have been demonstrated [22]. OH is considered an important fall risk factor and pathway in drug-related falls [23]. OH results in transient cerebral hypoperfusion upon standing, which may result in a syncope and/or fall. OH is a prevalent ADE in antidepressants, best known for TCAs, but also seen with SSRIs [7]. Furthermore, anticholinergic activity, which can increase fall risk, by among others risk of delirium and visual disturbances, increases with increasing plasma concentrations of nortriptyline (even at therapeutic levels) [24].

We observed a possible increased fall risk for users with higher plasma concentrations of TCA, which is in line with our hypothesis. However, the sample size was small. Furthermore, especially for amitriptyline the measured concentrations were below reference range for therapeutic effect or in therapeutic range, thus we cannot generalize our findings to potentially toxic concentrations. However, older adults are more at risk for adverse events due to altered pharmacokinetics and—dynamics [25]. TDM for TCAs is well-established in clinical practice, both for therapeutic effect and toxicity [26–29]. For SSRIs, the clinical use of TDM is less frequently employed, especially in older adults [30]. For most SSRIs therapeutic reference ranges are wide, evidence for a relationship between drug concentration and therapeutic outcome is weak and risk for toxicity is relatively low compared to TCAs [29, 31, 32]. So in general, available data do not suggest benefit for (routine) monitoring of SSRI plasma concentrations [31]. However, TDM for SSRIs is recommended in specific populations like advanced age [13, 32]. Since, studies have shown higher exposure to antidepressant concentrations compared to younger patients [33, 34]. And also adequate use of TDM has been shown to be cost effective in older adults [35, 36]. Thus, the lack of applying TDM and lack of lower blood concentrations as target, can possibly contribute to the risk of falls, an important ADE.

An important strength of our study is the prospective nature, using fall calendars. In falls research, this method is considered the golden standard to avoid recall bias [37]. Also, data on medication use were collected thoroughly, using both pharmacy prescription data and self-reported medication lists. Our study also has some important limitations. First, limited power was a problem. Despite the large B-PROOF database, the remaining group of participants that met the inclusion criteria was small. Due to small groups of antidepressant subclasses, we needed to pool the data and needed to standardize and categorize the plasma concentrations. Second, information about plasma concentrations during the fall incident was not available, since such an unplanned event cannot be anticipated for. Thus we only had blood samples from baseline and follow-up study visit. Baseline or follow-up plasma concentrations might not be representative for the plasma concentration at the time of the fall incidents. However, our main interest was to assess whether antidepressant plasma concentrations could serve as a predicting biomarker of future falls in clinical practice. In general, there is often only access to a single measurement if someone presents with a fall. Third, due to small sample size, not all analyses could be adjusted for confounders. In case of SSRIs, most of the confounders did not seem to influence the association between plasma concentration and falls. Thus it is likely that they would not be a major influence for the association between TCA plasma concentrations and falls. Fourth, for participants with undetectable plasma concentrations the respective concentration was set at half of the LLQ. Because participants were included based on prescription and self-reported medication usage data, the participants with undetectable concentrations were considered users. However, this does not guarantee actual usage. Lastly, our cohort consisted of relatively healthy community-dwelling older people. Frail older adults, can exhibit different patient characteristics, different pharmacokinetics which could lead to different results [25].

## Conclusion

Our explorative study showed a possible association between plasma concentrations and fall risk in older TCA users, but not in SSRI users. However, this needs to be interpreted with caution due to the small sample size and accompanying impossibility to perform multivariate analyses in this group. The current study has no direct clinical implications as our findings should be further re-evaluated in a larger cohort. Ideally replication would not only include multivariate assessment, but also include information about medication adherence and concentration measurements around the fall incident. However, this topic is important since it would give us more detailed insight in risk factors determining medication related fall risk and has the potential to personalize clinical decision making, maximizing benefit and minimizing harm in antidepressant use for older persons at risk of falling.

## Supplementary Information

Below is the link to the electronic supplementary material.

Supplementary file1 (DOCX 38 KB)

## Data Availability

Because of restrictions based on privacy regulations and informed consent of the participants, data cannot be made freely available in a public repository. B-PROOF data can be obtained upon request. Requests should be directed toward the principal investigators of the study, who have a protocol for approving data requests.
