# Supplementary material for: Are higher antidepressant plasma concentrations associated with fall risk in older antidepressant users?
Source: Eur Geriatr Med. 2023 Jan 19;14(1):89–97. doi: 10.1007/s41999-022-00742-1 (PMC9902404; doi:10.1007/s41999-022-00742-1)
Supplement: Supplementary file 1 — Supplementary file1 (DOCX 38 KB) [file 41999_2022_742_MOESM1_ESM.docx]

**Appendix S1. Additional information antidepressant plasma concentration**

To 100 µl of patients plasma, 750 µl of acetonitrile/methanol 42:8 (v/v%) containing the internal standard doxepin was added to precipitate proteins. Samples were vortexed, stored at -20°C for 10 minutes to optimize protein precipitation, vortexed again and centrifuged for 5 minutes at 2750 x g. Of the supernatant 5 µl was injected for amitriptyline, nortriptyline, citalopram and des(methyl)-citalopram and 10 µl was injected for fluoxetine, norfluoxetine, fluvoxamine and paroxetine onto a Thermo Scientific High Purity aquastar 50 X 2,1 mm, 5 µm column. A chromatographic gradient was applied using acetonitrile and water with a constant 5% addition of 3.5% (glacial acetic acid / 0.5% ammonium acetate in water. The flow rate was 0.4 ml/min and the column oven was kept at 30°C. Using multiple reaction monitoring (MRM), all antidepressants were measured in positive polarity mode as [M+H]^+^, using the mass transitions as shown in the table presented below.

**Mass transitions**

| Compound | Q1 (amu) | Q3 (amu) |
| --- | --- | --- |
| Amitriptyline | 278.25 | 233.35 |
| Nortriptyline | 264.25 | 233.25 |
| Citalopram | 325.15 | 109.05 |
| Des(methyl)-citalopram | 311.20 | 109.20 |
| Fluoxetine | 310.15 | 148.15 |
| Norfluoxetine | 296.10 | 134.10 |
| Fluvoxamine | 319.20 | 200.05 |
| Paroxetine | 330.10 | 192.15 |
| Doxepine | 280.15 | 107.05 |

The method was validated over a range of 22.0 -730 µg/L for amitriptyline, 22.0 - 733 µg/L for nortriptyline, 5.00 - 500 µg/L for citalopram, 5.00 - 500 µg/L for des-citalopram, 20.0 - 800 µg/L for fluoxetine, 20.0 - 800 µg/L for nor-fluoxetine, 20.0 - 800 µg/L for fluvoxamine, 5.00 – 200 µg/L for paroxetine, 2.00-200 µg/L for sertraline and 1.60-160 µg/L for Venlafaxine. For all the compounds accuracy ranged from 87.3% to 109.0%, intra-day imprecision was ≤ 11.3% and inter-day imprecision was ≤ 15%. The latter parameter was ≤ 17.6% for fluoxetine and ≤ 16.8% for paroxetine.

***Determination of semi-quantitative concentrations***

Semi-quantitative concentrations were determined for samples with levels below the LLQ for which the chromatographic peak had to signal to noise ratio greater than 3. Semi-quantitative results were calculated by extrapolating the existing standard curve.

**Table S1. Distribution of undetectable antidepressant plasma concentrations**

| Antidepressant ^a^ | No of concentrations | Number of undetectable plasma concentrations (%)^*^ |
| --- | --- | --- |
| Fluoxetine BSL  Fluoxetine FU | 6  6 | 1 (16.7)  1 (16.7) |
| Fluvoxamine BSL  Fluvoxamine FU | 3  3 | 0  0 |
| Sertraline BSL  Sertraline FU | 13  9 | 1 (7.7)  0 |
| Paroxetine BSL  Paroxetine FU | 47  35 | 8 (17.0)  2 (5.7) |
| (es)citalopram BSL  (es)citalopram FU | 13  9 | 2 (15.4)  0 |
| Venlafaxine BSL  Venlafaxine FU | 13  11 | 0  0 |
| Amitriptyline BSL^#^  Amitriptyline FU^#^ | 32  24 | 10 (31.3)  7 (29.2) |
| Nortriptyline BSL^#^  Nortriptyline FU^#^ | 9  3 | 0  0 |
| ^*^ Undetectable plasma concentrations were set at half of the LLQ  ^#^ For amitriptyline and nortriptyline semi-quantitative concentration values were determined. These are below LLQ and these were not considered as undetectable plasma concentrations. | | |

**Table S2. Concentration differences between fallers & non-fallers**

| Antidepressant ^a^ | No of concen-trations | Total concentration | Non-fallers | Fallers | P-value * |
| --- | --- | --- | --- | --- | --- |
| Fluoxetine BSL  Fluoxetine FU | 6  6 | 277.8 (78.7-352.5)  280.2 (113.4-323.6) | 348 (-)^#^  312.4 (-)^#^ | 98.3 (-)^#^  267.3 (-)^#^ | 0.2  0.7 |
| Fluvoxamine BSL  Fluvoxamine FU | 3  3 | 286 (-)  221 (-) | 131 (-) ^#^  126 (-) ^#^ | 308 (-)^#^  270.5 (-)^#^ | 1.0  1.0 |
| Sertraline BSL  Sertraline FU | 13  9 | 27 (22.5-43)  30 (24.5-48.5) | 37.5 (25.5-73)  56 (-) ^#^ | 25 (21-38)  30 (24.5-34.3) | 0.14  0.38 |
| Paroxetine BSL  Paroxetine FU | 47  35 | 53.2 (19.6-113)  53 (23.7-124) | 41.2 (16-113)  41.8 (9.6-124.5) | 58.1 (29.5-120.8)  77.8 (42.4-124) | 0.41  0.18 |
| (es)citalopram BSL  (es)citalopram FU | 13  9 | 63.9 (15.8-95.8)  67.9 (36.5-122.5) | 73.4 (18.8-105.3)  109.2 (-) ^#^ | 59.6 (15.8-95.8)  67.9 (28.6-110) | 0.71  0.67 |
| Venlafaxine BSL  Venlafaxine FU | 13  11 | 294.2 (175.9-409.7)  263.9 (152.5-469.3) | 333.1 (107-416.8)  482 (-)^#^ | 199.9 (175.9-425.9)  198.9 (124.7-403.9) | 1.0  0.33 |
| Amitriptyline BSL  Amitriptyline FU | 32  24 | 10.4 (4.9-25.4)  19.3 (4.9-40.8) | 10.4 (4.9-27.2)  29.5 (4.9-38.4) | 11 (4.9-22.7)  17.2 (4.9-44.2) | 0.78  0.84 |
| Nortriptyline BSL  Nortriptyline FU | 9  3 | 78.3 (9-147.5)  74.6 (-) ^#^ | 12 (-) ^#^  49.8 (-) ^#^ | 129 (37-165.3)  201.8 (-) ^#^ | 0.17  1.0 |
| ^a^ Concentrations presented as median with interquartile range (IQR) in μg/L  ^#^  IQR could not be calculated.  * statistically significant at p<0.05 | | | | | |

**Table S3. Therapeutic and (potential) toxic concentrations antidepressants**

|  | **Therapeutic (μg/L)** | **Potential toxic (μg/L)** |
| --- | --- | --- |
| Fluoxetine | 100-450 | 1500-2000 |
| Fluvoxamine | 50-250 | 650 |
| Sertraline | 50-300 | 300 |
| Paroxetine | 20-200 | 300 |
| (es)citalopram | 50-200 | 400-600 |
| Venlafaxine^a^ | 100-750 | >1000 |
| Amitriptyline | 100-300 | >400 |
| Nortriptyline | 50-150 | >250 |
| ^a^ Different ranges described; lowest & highest range combined.  From: https://tdm-monografie.org/ | | |

**Table S4. Association between SSRI plasma concentration at follow-up and fall risk prior study visit.**

|  | **N** | **Model 1 ^a^** | **P-value*** | **Model 2 ^b^** | **P-value*** |
| --- | --- | --- | --- | --- | --- |
| Concentration divided on median | 73 | 0.98 (0.34-2.82) | 0.98 | 1.13 (0.37-3.47) | 0.84 |
| Concentrations below LLQ & lowest tertile concentrations | 25 | Ref. | 0.11 | Ref. | 0.07 |
| Middle tertile | 24 | 3.73 (0.81-17.27) | 0.09 | 4.23 (0.88-20.31) | 0.07 |
| Highest tertile | 24 | 0.78 (0.23-2.60) | 0.68 | 0.68 (0.20-2.36) | 0.54 |
| Z-score | 73 | 0.80 (0.47-1.37) | 0.42 | - | - |
| Data is presented in Odds ratio with 95% confidence interval. N = number of plasma concentration samples. Number of events: 50  ^a^ Model 1 was adjusted for age and gender.  ^b^ Model 2 of category ‘divided on median’ was adjusted for MMSE. Model 2 of category ‘tertiles’ was adjusted for number of medication. For category ‘z-score’ no covariates influenced the model.  *statistically significant at p<0.05 | | | | | |

**Table S5. Association between TCA plasma concentration at follow-up visit and fall risk prior study visit.**

|  | **N** | **Unadjusted model** ^a^ | **P-value*** |
| --- | --- | --- | --- |
| Concentration divided on median | 27 | 0.65 (0.14-3.04) | 0.58 |
| Concentrations below LLQ & lowest tertile concentrations | 17 | Ref. |  |
| Middle & highest tertile | 10 | 2.07 (0.40-10.85) | 0.39 |
| Z-score | 27 | 1.04 (0.47-2.32) | 0.92 |
| Data is presented in Odds ratio with 95% confidence interval. N = number of plasma concentration samples; Number of events:16  ^a^ Adjusted models could not be created due to the limited amount of events.  *statistically significant at p<0.05 | | | |

**Table S6. Association between dosage (DDD) & (time to first) fall risk**

|  | **N** | **Crude model** | **P-value*** | **Model 1 ^a^** | **P-value*** |
| --- | --- | --- | --- | --- | --- |
| TCA | 40 | 1.38 (0.35-5.39) | 0.65 | 1.28 (0.32-5.19) | 0.73 |
| SSRI | 93 | 0.54 (0.27-1.07) | 0.08 | 0.56 (0.28-1.13) | 0.11 |
| Data is presented in Hazard ratio with 95% confidence interval. N = number of participants.  Number of events SSRI: 57; Number of events TCA: 23  TCA= Tricyclic Antidepressants; SSRI=Selective Serotonin Reuptake Inhibitor;  ^a^ Model 1 was adjusted for age and gender.  ^b^ Model 2: covariates did not influence the model  *statistically significant at p<0.05 | | | | | |
